# Risk of End-Stage Kidney Disease and Topiramate Use

**DOI:** 10.1016/j.ekir.2025.06.003

**Published:** 2025-06-10

**Authors:** Jean-Michel Halimi, Valentin Maisons, Jean-Baptiste de Fréminville, Sébastien Roger, Arnaud Bisson, Stéphanie Chadet, Laurent Fauchier

**Affiliations:** 1 Service de Néphrologie-Hypertension, Dialyse, Transplantation rénale, CHU de Tours, Tours, France; 2 INSERM UMR1327, ISCHEMIA, Université de Tours, Tours, France; 3 INI-CRCT, Tours, France; 4 Service de Cardiologie, Centre Hospitalier Universitaire Trousseau, Tours, France

**Keywords:** drug repurposing, nephroprotection, pharmacology, suicidality, therapeutics

## Abstract

**Introduction:**

Topiramate is widely used for migraine prevention but has pleiotropic effects on renal sodium reabsorption and inflammation. Whether these effects could be associated with lower risk of end-stage kidney disease (ESKD) and cardiovascular outcomes is unknown.

**Methods:**

Among 1,745,580 patients with a diagnosis of migraine, the risk of ESKD, cardiovascular outcomes, and death was assessed after propensity matching in patients treated with topiramate versus untreated patients.

**Results:**

Overall, 317,936 patients treated with topiramate were properly matched with 317,936 controls by age (43 years old), sex (women: 86%), clinical and biological parameters, comorbid conditions, and baseline medications. After a median follow-up of 3.5 years, patients receiving topiramate had a significant lower risk of ESKD (hazard ratio [HR]: 0.850; 95% confidence interval [CI]: 0.776–0.930; *P* < 0.0001). Among the subgroup of patients with available data, patients treated with topiramate had a smaller estimated glomerular filtration rate (eGFR) decline over time than the other patients. Albuminuria remained stable during follow-up in the topiramate group but increased in the other patients. They also had a lower risk of death (HR: 0.827 [0.799–0.856]), cardiac arrest or ventricular tachycardia/fibrillation (HR: 0.944 [0.898–0.992]), but a higher risk of ischemic stroke or thromboembolism (HR: 1.366 [1.318–1.416]) than other patients. Similar results were observed when men and women were analyzed separately. No association was found between topiramate use and risks of myocardial infarction, atrial fibrillation, or heart failure.

**Conclusion:**

In men and women with migraine, topiramate was significantly associated with lower risks of ESKD and death, but higher risks of ischemic stroke or thromboembolism.

## Graphical abstract

**Figure:**
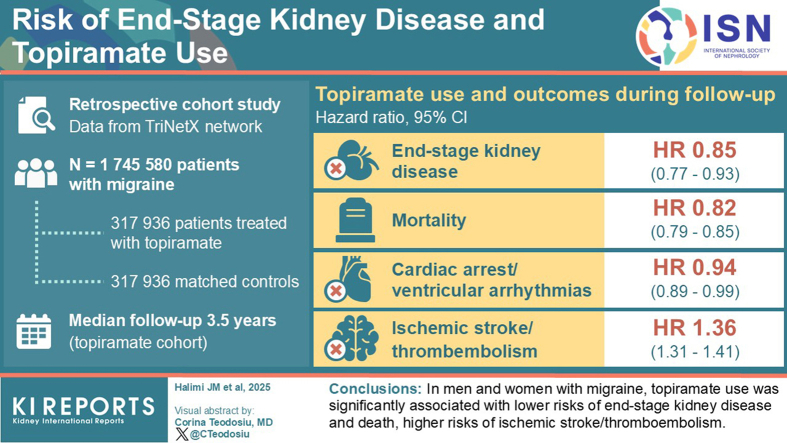

See Commentary on Page 2530

Topiramate is approved and widely used for the treatment of epilepsy and migraine prevention.^1^^,^^2^ More recently, it has also been shown that topiramate has antiinflammatory properties.^3^^,^^4^ Inflammation is recognized as a key factor for progression of chronic kidney diseases.^5^ Topiramate also affects sodium handling at the renal proximal tubular site by inhibiting carbonic anhydrase.^6^ Therefore, topiramate has effects on inflammation and renal proximal tubular sodium reabsorption, and in that respect, may be comparable to sodium-glucose cotransporter 2 inhibitors (SGLT2i) and glucagon-like peptide-1 receptor agonists (GLP1-RAs), which reduce the risk of ESKD and cardiovascular outcomes in many populations.^7^ Whether topiramate could offer similar benefits is presently unknown.

Interestingly, topiramate was shown to provide organ protection against ischemia-reperfusion in different organs in animal models, including in the kidneys, through beneficial effects on oxidative stress and apoptosis.^8^, ^9^, ^10^, ^11^ In addition, topiramate protected apoE-deficient mice from kidney damage in reducing foam cells glomerular accumulation and inflammatory markers’ expression.^12^ Topiramate reduced blood urea nitrogen levels in this model.^12^ Altogether, these data suggest that topiramate may have beneficial renal effects.

However, migraine may be associated with a higher risk of ischemic stroke and thromboembolism.^13^ It is thus necessary to study the effects of topiramate on ischemic stroke and thromboembolism.^13^ Finally, as stated in a recent review, “there is a paucity of data regarding sex differences in response to migraine prophylactic drugs… It should be a requirement that in migraine research, data are analyzed separately by sex”.^14^ Consequently, the potential effects of topiramate could be different in men and women, but this has not been specifically studied.

In the present study conducted in patients with a diagnosis of migraine, we retrospectively assessed the risk of ESKD as a primary end point, and cardiovascular events and death as secondary end points, in a large cohort of men and women treated by topiramate vs properly matched controls.

## Methods

### Patient Selection

The data used in this study came from the TriNetX network. It is a global federated health research network providing access to electronic medical records, including diagnoses, procedures, medications, laboratory values, and some genomic information for approximately 151 million deidentified patients across 126 large health care organizations in 17 countries worldwide. To have a homogenous group, patients included in the present analysis had a diagnosis of migraine (International Classification of Diseases 10th Revision code G43) and among them those receiving topiramate were compared with those not receiving topiramate (Supplementary Table S1). The analysis process included the following 2 main steps: (i) defining the cohorts through query criteria, and (ii) setting up and running the analysis. Setting up the analysis required definitions for the index event (i.e., topiramate treatment), outcomes criteria, and the time frame. Because some patients may be treated with the topiramate-phentermine combination for weight reduction purposes, patients treated with phentermine were excluded of the analysis.^15^ The query was run on the Global Collaborative Network; 124 health care organizations were queried and 124 health care organizations responded. This analysis included outcomes that occurred in the time period that started after the first occurrence of the index event (i.e., topiramate treatment) and ended 6 years after the first occurrence of the index event. Of note, the index date was aligned with the initiation of topiramate treatment. The index event only included events that occurred up to 20 years ago (Supplementary Table S2).

### Collected Data and Propensity Score Matching

The baseline parameters collected are presented in Table 1. They included demographics, race and ethnicity; body mass index; blood pressure; cardiovascular comorbidities; acute kidney failure and chronic kidney disease; diabetes mellitus; overweight and obesity; and noncardiovascular comorbidities, including among others, chronic obstructive pulmonary disease, personal history of nicotine dependence, alcohol abuse, cancers, Alzheimer’s disease, anxiety or stress-related mental disorders, and affective disorders. Laboratory parameters included cholesterol levels, eGFR using creatinine-based formula (Modification of Diet in Renal Disease equation), albuminuria, and HbA1c. Collected medication information included presence (but not the dose) of beta-blockers, calcium-channel blockers, angiotensin-converting enzyme inhibitors, angiotensin-receptor blockers, diuretics, antilipemic agents, medications used in diabetes mellitus, anticoagulants, and antiplatelet agents. Propensity score matching was performed on all collected parameters for the whole population (Supplementary Figure S1), and separately for men and women. The performance of the propensity score model was evaluated using standardized mean differences for all covariates. Before matching, several covariates had standardized mean differences > 0.1, indicating imbalance. After matching, all covariates showed standardized mean differences < 0.1, suggesting adequate balance between groups. The distribution of propensity scores also showed a substantial overlap between the 2 groups, supporting the robustness of the matching procedure.

**Table 1:** Baseline characteristics of patients before and after propensity score matching

Clinical characteristics	Before propensity-score matching				After propensity-score matching			
	Topiramate	No topiramate	*P*-value	Std diff. (%)	Topiramate	No topiramate	*P*-value	Std diff. (%)
	(*n* = 318,008)	(*n* = 1,487,579)			(*n* = 317,936)	(*n* = 317,936)		
Age at index (yrs), mean ± SD	42.2 ± 14.2	42.5 ± 16.0	< 0.001	2.2	42.2 ± 14.2	42.5 ± 14.9	< 0.001	2.4
Men, *n* (%)	45,052 (14.2%)	300,744 (20.2%)	< 0.001	16.1	45,052 (14.2%)	45,866 (14.4%)	0.004	0.7
White, *n* (%)	212,622 (66.9%)	970,615 (65.2%)	< 0.001	3.4	212,575 (66.9%)	210,172 (66.1%)	< 0.001	1.6
Black or African American, *n* (%)	41,541 (13.1%)	17,4011 (11.7%)	< 0.001	4.1	41,524 (13.1%)	39,436 (12.4%)	< 0.001	2
Hispanic or Latino, *n* (%)	23,981 (7.5%)	114,020 (7.7%)	0.017	0.5	23977 (7.5%)	24,941 (7.8%)	< 0.001	1.1
Asian, *n* (%)	6137 (1.9%)	55,281 (3.7%)	< 0.001	10.8	6137 (1.9%)	5962 (1.9%)	0.108	0.4
Systolic BP (mm Hg), mean ± SD	121.5 ± 16.7	121.9 ± 17.8	< 0.001	2.4	121.5 ± 16.7	121.9 ± 17.9	< 0.001	2.2
Diastolic BP (mm Hg), mean ± SD	75.0 ± 11.6	74.3 ± 12.2	< 0.001	5.3	75.0 ± 11.6	74.5 ± 12.4	< 0.001	3.9
Body mass index (kg/m^2^), mean ± SD	31.1 ± 8.4	28.7 ± 7.4	< 0.001	29.4	31.1 ± 8.4	30.5 ± 8.0	< 0.001	7.2
Comorbid conditions								
Hypertension, *n* (%)	75,548 (23.8%)	344,138 (23.1%)	< 0.001	1.5	75,512 (23.8%)	73,870 (23.2%)	< 0.001	1.2
Diabetes mellitus, *n* (%)	29,256 (9.2%)	116,219 (7.8%)	< 0.001	5	29,235 (9.2%)	28,427 (8.9%)	< 0.001	0.9
Smoker, *n* (%)	20,818 (6.5%)	96,537 (6.5%)	0.238	0.2	20,815 (6.5%)	20,321 (6.4%)	0.012	0.6
Overweight or obesity, *n* (%)	67,467 (21.2%)	219,646 (14.8%)	< 0.001	16.9	67,414 (21.2%)	66,025 (20.8%)	< 0.001	1.1
Dyslipidemia, *n* (%)	63,675 (20%)	298,989 (20.1%)	0.332	0.2	63,647 (20%)	62,133 (19.5%)	< 0.001	1.2
Alcohol related diagnoses, *n* (%)	6177 (1.9%)	23,132 (1.6%)	< 0.001	3	6174 (1.9%)	6179 (1.9%)	0.964	0
Cardiovascular comorbidities								
Heart failure, *n* (%)	7318 (2.3%)	31,590 (2.1%)	< 0.001	1.2	7315 (2.3%)	6968 (2.2%)	0.003	0.7
Coronary artery disease, *n* (%)	13,104 (4.1%)	62,842 (4.2%)	0.008	0.5	13,102 (4.1%)	12,833 (4%)	0.088	0.4
Myocardial infarction, *n* (%)	2768 (0.9%)	14,046 (0.9%)	< 0.001	0.8	2768 (0.9%)	3445 (1.1%)	< 0.001	2.2
Dilated cardiomyopathy, *n* (%)	521 (0.2%)	2465 (0.2%)	0.813	0	521 (0.2%)	500 (0.2%)	0.511	0.2
Ischemic stroke, *n* (%)	9441 (3%)	34,527 (2.3%)	< 0.001	4	9433 (3%)	9077 (2.9%)	0.008	0.7
Intracranial hemorrhage, *n* (%)	1498 (0.5%)	5158 (0.3%)	< 0.001	1.9	1498 (0.5%)	1299 (0.4%)	< 0.001	0.9
Valve disease, *n* (%)	6708 (2.1%)	32,396 (2.2%)	0.016	0.5	6706 (2.1%)	6510 (2%)	0.085	0.4
Mitral regurgitation, *n* (%)	5547 (1.7%)	25,935 (1.7%)	0.973	0	5545 (1.7%)	5248 (1.7%)	0.004	0.7
Mitral stenosis, *n* (%)	941 (0.3%)	4643 (0.3%)	0.135	0.3	940 (0.3%)	1017 (0.3%)	0.081	0.4
Aortic regurgitation, *n* (%)	2187 (0.7%)	11,668 (0.8%)	< 0.001	1.1	2183 (0.7%)	2422 (0.8%)	< 0.001	0.9
Aortic stenosis, *n* (%)	1745 (0.5%)	9713 (0.7%)	< 0.001	1.3	1744 (0.5%)	1987 (0.6%)	< 0.001	1
Atrial fibrillation or flutter, *n* (%)	5259 (1.7%)	29612 (2%)	< 0.001	2.5	5259 (1.7%)	5131 (1.6%)	0.205	0.3
Sinus node disease, *n* (%)	2559 (0.8%)	7681 (0.5%)	< 0.001	3.6	2559 (0.8%)	1888 (0.6%)	< 0.001	2.5
Atrioventricular and/or LBBB, *n* (%)	3337 (1%)	15,888 (1.1%)	0.351	0.2	3334 (1%)	3553 (1.1%)	0.008	0.7
Left BBB, *n* (%)	771 (0.2%)	4244 (0.3%)	< 0.001	0.8	771 (0.2%)	913 (0.3%)	0.001	0.9
Right BBB, *n* (%)	2199 (0.7%)	10,410 (0.7%)	0.61	0.1	2198 (0.7%)	2317 (0.7%)	0.076	0.4
Previous pacemaker, *n* (%)	1369 (0.4%)	5949 (0.4%)	0.014	0.5	1369 (0.4%)	1223 (0.4%)	0.004	0.7
Previous ICD, *n* (%)	794 (0.2%)	3315 (0.2%)	0.004	0.6	794 (0.2%)	785 (0.2%)	0.821	0.1
Noncardiovascular comorbidities								
Kidney disease, *n* (%)	13,711 (4.3%)	62,990 (4.2%)	0.05	0.4	13709 (4.3%)	13,490 (4.2%)	0.175	0.3
COPD, *n* (%)	10,389 (3.3%)	40,020 (2.7%)	< 0.001	3.4	10384 (3.3%)	10,092 (3.2%)	0.038	0.5
Sleep apnea syndrome, *n* (%)	33,737 (10.6%)	102,934 (6.9%)	< 0.001	13.1	33,684 (10.6%)	32,931 (10.4%)	0.002	0.8
Peripheral vascular disease, *n* (%)	3239 (1%)	14,516 (1%)	0.027	0.4	3237 (1%)	3378 (1.1%)	0.081	0.4
Previous cancer, *n* (%)	56,826 (17.9%)	255,818 (17.2%)	< 0.001	1.8	56,800 (17.9%)	56,097 (17.6%)	0.021	0.6
Thyroid diseases, *n* (%)	43,640 (13.7%)	183,766 (12.4%)	< 0.001	4.1	43,620 (13.7%)	43,899 (13.8%)	0.31	0.3
Amyloidosis, *n* (%)	195 (0.1%)	999 (0.1%)	0.245	0.2	195 (0.1%)	202 (0.1%)	0.725	0.1
Malnutrition, *n* (%)	3631 (1.1%)	17,506 (1.2%)	0.096	0.3	3629 (1.1%)	4263 (1.3%)	< 0.001	1.8
Cognitive impairment, *n* (%)	264 (0.1%)	1703 (0.1%)	< 0.001	1	264 (0.1%)	265 (0.1%)	0.965	0
Anxiety, *n* (%)	107,486 (33.8%)	410,000 (27.6%)	< 0.001	13.6	107,432 (33.8%)	104,842 (33%)	< 0.001	1.7
Depression / mood disorders, *n* (%)	100,972 (31.8%)	340,140 (22.9%)	< 0.001	20	100,912 (31.7%)	97,815 (30.8%)	< 0.001	2.1
Biological characteristics								
Total cholesterol (mg/dl), mean ± SD	185.0 ± 42.2	185.2 ± 43.0	0.201	0.4	185.0 ± 42.2	184.8 ± 43.0	0.233	0.5
LDL cholesterol (mg/dl), mean ± SD	107.6 ± 35.0	107.3 ± 35.4	0.016	0.8	107.6 ± 35.0	107.2 ± 35.6	0.009	1.1
HDL cholesterol (mg/dl), mean ± SD	51.6 ± 17.4	53.6 ± 18.4	< 0.001	11.1	51.6 ± 17.4	52.2 ± 18.0	< 0.001	3.7
Triglyceride (mg/dl), mean ± SD	136.6 ± 116.5	128.4 ± 116.2	< 0.001	7.1	136.6 ± 116.5	134.2 ± 110.4	< 0.001	2.1
Hemoglobin A1c (%), mean ± SD	6.0 ± 1.6	6.0 ± 1.8	< 0.001	3.7	6.0 ± 1.6	6.0 ± 1.7	< 0.001	4.4
Estimated GFR (MDRD, ml/min), mean ± SD	87.5 ± 25.1	89.1 ± 27.2	< 0.001	6	87.5 ± 25.1	88.3 ± 26.6	< 0.001	3
Albuminuria (mg/g), mean ± SD	92.1 ± 412.9	125.8 ± 598.5	0.012	6.6	92.1 ± 412.9	130.3 ± 703.9	0.028	6.6
Albuminuria 0–30 mg/g, *n* (%)	1793 (0.6%)	7722 (0.5%)	0.002	0.6	1793 (0.6%)	1758 (0.6%)	0.556	0.1
Albuminuria 30–300 mg/g, *n* (%)	508 (0.2%)	2223 (0.1%)	0.175	0.3	508 (0.2%)	502 (0.2%)	0.85	0
Albuminuria > 300 mg/g, *n* (%)	150 (0%)	812 (0.1%)	0.1	0.3	150 (0%)	159 (0.1%)	0.609	0.1
Treatments								
Beta blockers, *n* (%)	79,195 (24.9%)	250,276 (16.8%)	< 0.001	20	79,124 (24.9%)	81,324 (25.6%)	< 0.001	1.6
Calcium channel blockers, *n* (%)	40,299 (12.7%)	128,446 (8.6%)	< 0.001	13.1	40,256 (12.7%)	40,962 (12.9%)	0.008	0.7
ACE inhibitors, *n* (%)	30,998 (9.7%)	102,662 (6.9%)	< 0.001	10.3	30,959 (9.7%)	30,536 (9.6%)	0.073	0.5
Angiotensin 2 inhibitors, *n* (%)	20,536 (6.5%)	73,561 (4.9%)	< 0.001	6.5	20,514 (6.5%)	20,548 (6.5%)	0.862	0
Digitalis glycosides, *n* (%)	831 (0.3%)	3672 (0.2%)	0.138	0.3	830 (0.3%)	871 (0.3%)	0.32	0.2
Diuretics, *n* (%)	55,210 (17.4%)	162186 (10.9%)	< 0.001	18.6	55,144 (17.3%)	55,513 (17.5%)	0.222	0.3
Lipid lowering drugs, *n* (%)	60,245 (18.9%)	197094 (13.2%)	< 0.001	15.5	60,178 (18.9%)	60,299 (19%)	0.699	0.1
Glucose-lowering therapy, *n* (%)	41,284 (13%)	121,836 (8.2%)	< 0.001	15.6	41,226 (13%)	39,928 (12.6%)	< 0.001	1.2
Insulin, *n* (%)	21,046 (6.6%)	71,299 (4.8%)	< 0.001	7.9	21,036 (6.6%)	20,529 (6.5%)	0.01	0.6
Noninsulin glucose-lowering therapy, *n* (%)	30,790 (9.7%)	81,845 (5.5%)	< 0.001	15.8	30,734 (9.7%)	29,109 (9.2%)	< 0.001	1.8
Metformin, *n* (%)	23,831 (7.5%)	63,381 (4.3%)	< 0.001	13.8	23,791 (7.5%)	22,993 (7.2%)	< 0.001	1
Sulfonylureas, *n* (%)	4706 (1.5%)	15,494 (1%)	< 0.001	3.9	4700 (1.5%)	4812 (1.5%)	0.247	0.3
GLP-1 receptor agonists, *n* (%)	7221 (2.3%)	17,135 (1.2%)	< 0.001	8.6	7203 (2.3%)	6783 (2.1%)	< 0.001	0.9
DPP4 inhibitors, *n* (%)	3179 (1%)	10,210 (0.7%)	< 0.001	3.4	3178 (1%)	3051 (1%)	0.106	0.4
SGLT2 inhibitors, *n* (%)	1888 (0.6%)	6650 (0.4%)	< 0.001	2	1888 (0.6%)	1817 (0.6%)	0.242	0.3
Thiazolidinediones, *n* (%)	836 (0.3%)	2044 (0.1%)	< 0.001	2.8	835 (0.3%)	649 (0.2%)	< 0.001	1.2
Antiplatelet therapy, *n* (%)	52,602 (16.5%)	181,173 (12.2%)	< 0.001	12.5	52,560 (16.5%)	53,324 (16.8%)	0.01	0.6
Anticoagulant, *n* (%)	7236 (2.3%)	29,895 (2%)	< 0.001	1.8	7230 (2.3%)	6998 (2.2%)	0.049	0.5
Long-term use of NSAID, *n* (%)	5537 (1.7%)	22,005 (1.5%)	< 0.001	2.1	5536 (1.7%)	5292 (1.7%)	0.018	0.6

BBB, bundle branch block; BP, blood pressure; COPD, chronic obstructive pulmonary disease; DPP4, dipeptidyl peptidase 4; GFR, glomerular filtration rate; GLP-1, glucagon-like peptide-1; HDL, high-density lipoprotein; ICD, implantable cardioverter-defibrillator; LBBB, left bundle branch block; LDL, low-density lipoprotein; MDRD, Modification of Diet in Renal Disease equation; NSAID, nonsteroidal antiinflammatory drug; SGLT2, sodium-glucose cotransporter 2; Std diff., standard mean difference.

### Statistical Analyses and Outcome Measures

The results are presented with mean and SD or median and interquartile range (IQR) for quantitative parameters and number and percentages for qualitative parameters. Kaplan-Meier analysis estimates probability of outcomes was used. A Log rank test was used to compare the risk of these events during follow-up in the patients receiving topiramate and in those who did not.^16^

Incidence of ESKD (defined as chronic dialysis or renal transplantation), as a primary outcome, death and cardiovascular events, as secondary outcomes, were assessed over a maximum follow-up of 6 years. For all analyses, we first studied the whole population and then men and women, separately. In the subgroup of patients with eGFR or albuminuria both at baseline and during follow-up, the changes in eGFR and in albuminuria during follow-up were compared among inpatients in the topiramate group and the non-topiramate group.

Given the large size of the population, significant associations between topiramate use and events could be chance results, despite proper propensity score matching and multiple adjustments. Therefore, 2 outcomes probably not affected by topiramate (presbyopia and cataract surgery) were selected and used as “neutral events” for falsification analysis. In addition, we proposed to classify the reliability of the associations between topiramate and outcomes during follow-up as “low” when the point estimate of the HR difference was < 5%, “moderate” when it was 6% to 10%, and “high” when it was > 10%.

## Results

### Baseline Characteristics

Overall, 1,805,587 patients had a diagnosis of migraine: 318,008 patients treated with topiramate and 1,487,579 patients not treated with topiramate. These 2 groups were different for most clinical diagnoses, race, ethnicity, sex ratio, blood pressure, body mass index, comorbid conditions, biological results, and treatments (Table 1).

### Analysis After Propensity Matching

After propensity score matching in the whole population, 317,936 patients treated with topiramate were compared with 317,936 patients without topiramate (Table 1 and Supplementary Figure S2). These 2 groups were well-matched (standard difference of all parameters between the 2 groups < 10%) on baseline characteristics (Table 1). In these 2 groups, the mean age was 43 years and men represented 14% of the patients (Table 1). Patients were similar with regard to race and ethnicity, body mass index, systolic and diastolic blood pressure, comorbid conditions, smoking habits, depression or mood disorders, and treatments (Table 1). They were also well-matched for low-density lipoprotein cholesterol and high-density cholesterol levels, triglyceride levels, hemoglobin A1c, eGFR, and albuminuria (Table 1). The use of antihypertensive or other cardiovascular medications, glucose-lowering therapy, anticoagulants and antiplatelet therapy was well-matched (Table 1).

Propensity score matching was also performed in men and women separately. After this procedure, men were well-matched (standard difference of all parameters between the 2 groups < 10%) on baseline characteristics (Supplementary Table S3 and Supplementary Figure S3). The same findings were observed for women (Supplementary Table S4 and Supplementary Figure S4).

### Topiramate and Major Outcomes During Follow-Up

#### Analysis of the Whole Population

**Figure 1:**
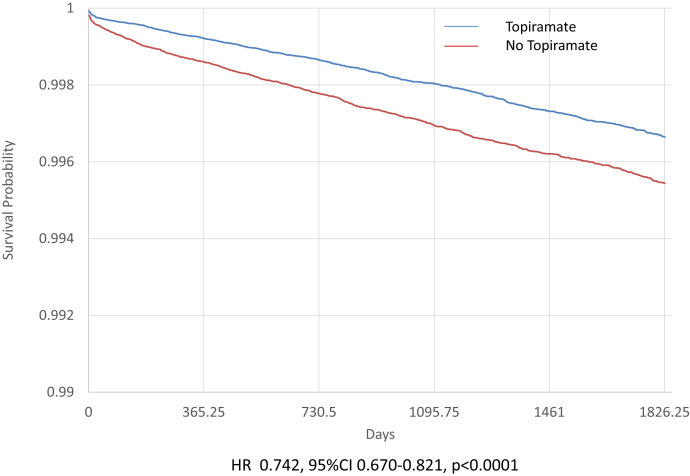
Event-free curve for acute or chronic dialysis or renal transplantation in matched patients treated with topiramate (blue) or not treated with topiramate (red). CI, confidence interval; HR, hazard ratio.

The median follow-up was 3.5 (3.7 [IQR: 4.8] and 3.3 [IQR: 5.0]) years in patients treated with topiramate and in those without, respectively). Patients receiving topiramate had a significant lower risk of acute or chronic dialysis or renal transplantation in the unmatched population (Supplementary Table S5) and in the matched population (yearly rate: 0.10% vs. 0.13%, HR: 0.742 [95% confidence interval: 0.670–0.821]) (Figure 1) and chronic dialysis or renal transplantation (HR: 0.850 [0.776–0.930], *P* < 0.0001) than the other patients (Table 2). The changes in eGFR during follow-up were small but patients in the topiramate group had a smaller eGFR decline than the other patients. Albuminuria remained stable during follow-up in the topiramate group but increased in the other patients (Supplementary Table S6).

**Table 2:** Clinical outcomes during follow-up in the matched population (3.5 ± 2.2 yrs)

Events	Topiramate		No topiramate		Hazard ratio (95% CI)	*P* value
	(*n* = 317,936)		(*n* = 317,936)			
	Number of events	Yearly rate, %	Number of events	Yearly rate, %		
Death^a^	7089	0.57	8258	0.72	0.758 (0.734–0.782)	< 0.0001
Chronic dialysis or renal transplantation^a^	922	0.08	962	0.09	0.850 (0.776–0.930)	< 0.0001
Acute MI	3688	0.31	3238	0.31	0.999 (0.953–1.047)	0.97
Ischemic stroke or thromboembolism^a^	7708	0.63	5367	0.49	1.285 (1.241–1.331)	< 0.0001
VT/VF/cardiac arrest	5049	0.42	4443	0.42	1.003 (0.963–1.044)	0.9
Hosp. for HF	3609	0.28	3281	0.28	0.981 (0.936–1.029)	0.44
MI/stroke/HF/death	29,450	2.16	26840	2.10	0.992 (0.976–1.009)	0.35

CI, confidence intervals; HF, heart failure; MI, myocardial infarction; VT/VF, ventricular tachycardia or ventricular fibrillation.

a Associations with topiramate considered as “high reliable” associations (i.e., point estimate of the hazard ratio difference > 10%, cf Methods section).

**Figure 2:**
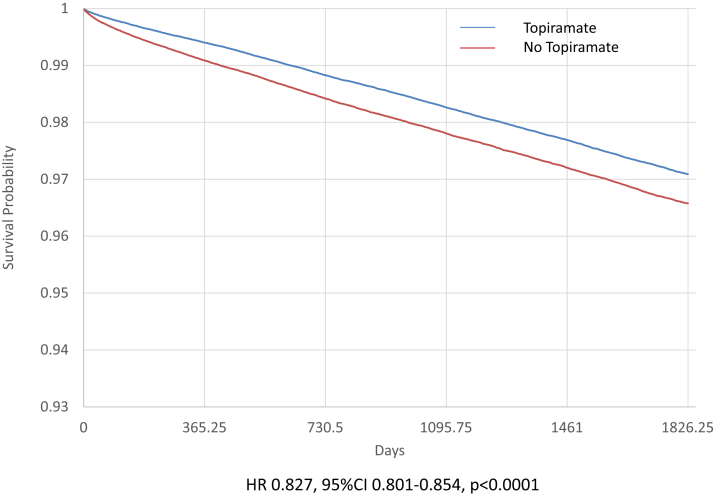
Event-free curve for all-cause death in matched patients treated with topiramate (blue) or not treated with topiramate (red). CI, confidence interval; HR, hazard ratio.

Patients receiving topiramate had a significant lower risk of death (yearly rate: 0.91% vs. 1.06%, HR: 0.827 [95% confidence interval: 0.799–0.856]) (Table 2). The difference between the 2 groups for the risk of death started rapidly after topiramate exposure (Figure 2). The risk was lower for cardiac arrest or ventricular tachycardia or fibrillation (HR: 0.944 [0.898–0.992]) (Table 2). Topiramate was associated with a higher risk of ischemic stroke or thromboembolism (yearly rate: 1.08% vs. 0.78%, HR: 1.366 [1.318–1.416]) (Table 2). Of note, the HR difference of presbyopia (HR: 1.056 [HR: 1.027–1.087) and cataract surgery (HR: 0.915 [HR: 0.847–0.987) risks (used as falsification analysis) between the 2 groups did not exceed 10%; and therefore, the reliability of these associations was considered “low or moderate.”

#### Separate Analysis in Men and in Women

The median follow-up was 3.5 (IQR: 4.6) years in men and 3.6 (IQR: 4.6) years in women. Findings observed in the whole population were confirmed when men and women were analyzed separately. As shown in Table 3, Table 4 and 4, both men and women who were treated with topiramate had a significant lower risk of death, dialysis, or renal transplantation; and a significant higher risk of ischemic stroke/thromboembolism than men and women who were not treated with topiramate.

**Table 3:** Outcomes in men treated with topiramate versus other men after propensity score matching

Events	Topiramate		No topiramate		Hazard ratio (95% CI)
	*n* = 41,503		*n* = 41,503		
	Number of events	Yearly rate, %	Number of events	Yearly rate, %	
Acute/chronic dialysis or renal transplantation^a^	159	0.18	210	0.23	0.681 (0.554–0.837)
Death	1428	1.55	1448	1.70	0.882 (0.820–0.949)
Acute MI	804	0.88	699	0.85	1.027 (0.928–1.136)
Ischemic stroke or thromboembolism^a^	1438	1.54	947	1.13	1.387 (1.277–1.505)
VT/VF/cardiac arrest	749	0.76	729	0.80	0.930 (0.840–1.030)
Hosp. for HF	503	0.47	460	0.46	1.004 (0.884–1.139)

CI, confidence intervals; ESKD, end-stage kidney disease; HF, heart failure; Hosp., hospitalization; MI, myocardial infarction; VT/VF, ventricular tachycardia or ventricular fibrillation.

a Associations with topiramate considered as “high reliable” associations (i.e., point estimate of the hazard ratio difference > 10%, cf Methods section).

**Table 4:** Outcomes in women treated with topiramate vs other women after propensity score matching

Events	Topiramate		No Topiramate		Hazard ratio (95% CI)
	*n* = 247,253		*n* = 247,253		
	Number of events	Yearly rate, %	Number of events	Yearly rate, %	
Acute/chronic dialysis or renal transplantation^a^	568	0.09	692	0.12	0.731 (0.654–0.817)
Death^a^	4898	0.82	5173	0.94	0.838 (0.806–0.871)
Acute MI	2700	0.46	2331	0.44	1.023 (0.968–1.081)
Ischemic stroke or thromboembolism^a^	6115	1.00	4081	0.74	1.347 (1.294–1.401)
VT/VF/Cardiac arrest	2372	0.38	2226	0.38	0.954 (0.901–1.011)
Hosp. for HF	1857	0.27	1604	0.25	1.058 (0.989–1.131)

CI, confidence intervals; ESKD, end-stage kidney disease; HF, heart failure; Hosp., hospitalization; MI, myocardial infarction; VT/VF, ventricular tachycardia or ventricular fibrillation.

a Associations with topiramate considered as “high reliable” associations (i.e., point estimate of the hazard ratio difference > 10%, cf Methods section).

## Discussion

In the present study of 635,872 patients with a diagnosis of migraine, topiramate use was significantly associated with lower risks of death and ESKD, less decline in eGFR, and less albuminuria, but a higher risk of ischemic stroke or thromboembolism in men and women.

Topiramate use was associated with a lower risk of ESKD, and the association was observed in men and women, independent of history of blood pressure, diabetes mellitus, treatments for kidney and cardiovascular disease (including renin-angiotensin system blockers, SGLT2i and GLP1-RA), HbA1c, lipids, eGFR levels, and albuminuria (although the number of patients with albuminuria results was small). Of note, the use of nonsteroidal antiinflammatory drug was similar in subjects treated with topiramate and in the other subjects, even before propensity matching. The magnitude of this association was of clinical interest and was classified as a “high reliability” association. The exact mechanism is unknown. However, topiramate reduces the proximal tubular reabsorption of sodium by inhibiting carbonic anhydrase (the same mechanism associated with acetazolamide).^6^ It is conceivable that its use results in an increased sodium load to the macula densa. Then, this sodium load could activate the tubulo-glomerular feedback^17^ resulting in lower glomerular capillary pressure. Because SGLT2i and GLP1-RAs also affect sodium reabsorption in the renal proximal tubules and offer substantial nephroprotection to patients with CKD, it is tempting to speculate that some of the renal protection provided by SGLT2i and GLP1-RAs is due to the reduced sodium reabsorption at the renal proximal tubule level. However, other mechanisms may be implicated. Proinflammatory cytokines are also mediators of CKD progression.^5^ Interestingly, topiramate was shown to reduce kidney damage in a rat model of renal ischemia by decreasing tumor necrosis factor-α and interleukin-1 beta levels.^8^ Of note, inflammation increases the number of gamma-aminobutyric acid receptors, at least in the brain,^18^ and topiramate interacts with these receptors. These receptors have been identified in the human renal tubular cells; however, whether they mediate renal inflammation has not been studied.^19^

The association between topiramate use and cardiac arrest or ventricular tachycardia or fibrillation was significant but modest. We classified it as a “low reliable” association. However, this association was independent of baseline characteristics because the 2 groups as they, were well-balanced. Furthermore, in experimental models, topiramate prevented the cardiotoxicity of doxorubicin, improved the serum levels of cardiac biomarkers, decreased oxidative stress, and reduced fibrosis.^20^ If confirmed, this action of topiramate is unlikely to be related to a favorable effect on atherosclerosis because the incidence of myocardial infarction was not lower with topiramate use and the risk of ischemic stroke or thromboembolism was even higher in patients treated with topiramate. However, it may be related to its effects on voltage-gated sodium channels that may play an important role in the initiation of ventricular arrhythmias.^21^ Some pathogenic SCN5A variants causing Brugada syndrome were recently associated to a markedly increase in the risk of ventricular tachycardia or ventricular fibrillation^22^^,^^23^ and it has been reported that topiramate may reveal or worsen Brugada syndrome.^24^ Whether the lower risk of ventricular arrhythmias with topiramate is partly related to its effect on voltage-gated sodium channels is unknown but it could be related to its effect on inflammation.

Interestingly, topiramate demonstrated protective effects against organ damage in experimental models of ischemia reperfusion injury.^25^, ^26^, ^27^ However, whether these results may have relevance to our findings is unknown.

Our study has several strengths. First, this is likely the largest study worldwide assessing the effect of topiramate on hard outcomes. Second, the analysis of this study was restricted to patients with a diagnosis of migraine but not epilepsy to have a homogenous cohort. Third, patients from both groups were well-matched on all available parameters, including demographics, blood pressure, body mass index, race and ethnicity, comorbid conditions, biological parameters, and medications used. Fourth, there was no meaningful effect of topiramate on the risk of presbyopia or cataract surgery; these outcomes were used to assess internal validity.

Our study has also some limitations. Whether the results would be similar in patients treated with topiramate for other conditions such as epilepsy is unknown. Patients in our study were those having medical encounters with health care systems contributing to the research network. However, the exposure and comparison groups were drawn from the same database over the same period and with the same duration of follow-up, thus minimizing the risk of bias. Our results need to be validated in other studies. In the present study, it was not possible to determine the duration of topiramate treatment.

The study used administrative coding for diagnoses but other parameters that were not administrative by nature were included in the analysis, including blood pressure, body mass index, high-density lipoprotein cholesterol and low-density lipoprotein cholesterol, triglycerides, eGFR, albuminuria (although the number of patients with albuminuria results was small), HbA1c, and medications used. Although we controlled for an extensive list of variables, some biases might not have been fully eliminated. No causal inferences can be drawn because our study was not a randomized clinical trial. Our findings suggest but do not demonstrate that topiramate reduces the risk of ESKD, cardiovascular outcomes, and death, although the absence of a meaningful association of topiramate with “neutral” events suggests that our methodology is unlikely to be markedly flawed. We hypothesized that topiramate beneficial effects could be due, at least partly, to reduction of inflammation, but no direct measure of inflammation was available.

Our results strongly suggest that topiramate may be associated with a lower risk of ESKD and death, and that the magnitude of these effects was clinically meaningful. If confirmed, our findings implicate that future trials focused on these outcomes evaluating topiramate versus placebo should be planned before it can be repurposed as a nephroprotective medication, as has been done for SGLT2i and GLP1-RA.^28^, ^29^, ^30^ Interestingly, the incidence of CKD was higher in patients with migraine than in those without migraine in a nationwide population-based cohort study^31^ but not in all studies.^32^ In our study, all patients had a diagnosis of migraine, and it is therefore possible that the incidence of CKD in this population is higher than in the general population.

In conclusion, topiramate was associated with a reduced relative risk of ESKD in this retrospective study. These results may provide some insight into the mechanisms of organ protection associated with SGLT2 inhibitors and GLP1-RAs, because the association between topiramate and the risk of ESKD was not observed for risk of heart failure. This observation suggests that these cardiac and renal effects SGLT2 inhibitors and GLP1-RAs could be dissociated, and that their underlying mechanisms might be distinct. However, topiramate was associated with a 37% increased relative risk of ischemic stroke or thromboembolism in men and women.

## Disclosure

All the authors declared no competing interests.

## Author Contributions

JMH and LF contributed the study conception, statistical analysis, writing of the first draft, and access to the data. VM, JBdF, SR, AB, and SC contributed to corrections of the drafts and interpretation of data. All the authors contributed to drafting and correcting of the manuscript.

## Supplementary Material

Supplementary File (PDF) **Figure S1.** Covariate balance before and after propensity score matching (PSM). **Figure S2.** Propensity score density function in all patients - before (left panel) and after matching (right panel). **Figure S3.** Propensity score density function in men - before (left panel) and after matching (right panel). **Figure S4.** Propensity score density function in women - before (left panel) and after matching (right panel). **Table S1.** Cohorts’ definition. **Table S2.** Outcomes definition. **Table S3.** Baseline characteristics in men before and after propensity score matching. **Table S4.** Baseline characteristics in women before and after propensity score matching. **Table S5.** Clinical outcomes during follow-up in the unmatched population (3.4 ± 2.3 years). **Table S6.** Summary of GFR and albuminuria changes (for patients having both baseline and 24 to 36 months FU evaluations) with paired and independent *t* test results. Strobe Checklist.

## Footnotes

Supplementary File (PDF)
**Figure S1.** Covariate balance before and after propensity score matching (PSM). **Figure S2.** Propensity score density function in all patients - before (left panel) and after matching (right panel). **Figure S3.** Propensity score density function in men - before (left panel) and after matching (right panel). **Figure S4.** Propensity score density function in women - before (left panel) and after matching (right panel). **Table S1.** Cohorts’ definition. **Table S2.** Outcomes definition. **Table S3.** Baseline characteristics in men before and after propensity score matching. **Table S4.** Baseline characteristics in women before and after propensity score matching. **Table S5.** Clinical outcomes during follow-up in the unmatched population (3.4 ± 2.3 years). **Table S6.** Summary of GFR and albuminuria changes (for patients having both baseline and 24 to 36 months FU evaluations) with paired and independent *t* test results. Strobe Checklist.
