# Supplementary material for: Risk of End-Stage Kidney Disease and Topiramate Use
Source: Kidney Int Rep. 2025 Jun 10;10(8):2642–50. doi: 10.1016/j.ekir.2025.06.003 (PMC12347801; doi:10.1016/j.ekir.2025.06.003)
Supplement: Supplementary File (PDF) — Figure S1. Covariate balance before and after propensity score matching (PSM). Figure S2. Propensity score density function in all patients - before (left panel) and after matching (right panel). Figure S3. Propensity score density function in men - before (left panel) and after matching (right panel). Figure S4. Propensity score density function in women - before (left panel) and after matching (right panel). Table S1. Cohorts’ definition. Table S2. Outcomes definition. Table S3. Baseline characteristics in men before and after propensity score matching. Table S4. Baseline characteristics in women before and after propensity score matching. Table S5. Clinical outcomes during follow-up in the unmatched population (3.4 ± 2.3 years). Table S6. Summary of GFR and albuminuria changes (for patients having both baseline and 24 to 36 months FU evaluations) with paired and independent t test results. Strobe Checklist. [file mmc1.pdf]

Supplemental material

Supplemental Table 1 – Cohorts' Definition

This section lists all terms used in the definitions of the two cohorts.

Query Criteria for Cohort 1 (query name: Migraine, Topiramate, No Phentermine)

Patients must have:
- all of the following:
  - Migraine (UMLS:ICD10CM:G43) (at least 18 years old at event); and
  - Medications: VA classes (NLM:VA:VA000); and
  - BMI (TNX:9083) ((most recent occurrence)); and
  - topiramate (NLM:RXNORM:38404) (at least 18 years old at event).

Patients cannot have:
- phentermine (NLM:RXNORM:8152).

Query Criteria for Cohort 2 (query name: Migraine, No Topiramate, No Phentermine)

Patients must have:
- all of the following:
  - Migraine (UMLS:ICD10CM:G43) (at least 18 years old at event); and
  - Medications: VA classes (NLM:VA:VA000); and
  - BMI (TNX:9083) ((most recent occurrence)).

Patients cannot have:
- any of the following:
  - phentermine (NLM:RXNORM:8152); or
  - topiramate (NLM:RXNORM:38404) (at least 18 years old at event).

Supplemental Table 2 – Outcomes Definition

This analysis includes the following outcomes:

Death

Patients must have:

any of the following:

Deceased (Deceased); or

Ill-defined and unknown cause of mortality (UMLS:ICD10CM:R99).

Ischemic stroke or thromboembolism

Patients must have:

any of the following:

Cerebral infarction (UMLS:ICD10CM:I63); or

Arterial embolism and thrombosis (UMLS:ICD10CM:I74).

Acute MI

Patients must have:

Acute myocardial infarction (UMLS:ICD10CM:I21).

ICD implantation

Patients must have:

any of the following:

Insertion or replacement of permanent implantable defibrillator system, with transvenous lead(s), single or dual chamber (UMLS:CPT:33249); or

Presence of automatic (implantable) cardiac defibrillator (UMLS:ICD10CM:Z95.810); or

Insertion of implantable defibrillator pulse generator only; with existing single lead (UMLS:CPT:33240).

VT/VF/Cardiac arrest

Patients must have:

any of the following:

Cardiac arrest (UMLS:ICD10CM:I46); or

Ventricular fibrillation (UMLS:ICD10CM:I49.01); or

Ventricular fibrillation and flutter (UMLS:ICD10CM:I49.0); or

Ventricular tachycardia (UMLS:ICD10CM:I47.2).

Cardiac arrest

Patients must have:

Cardiac arrest (UMLS:ICD10CM:I46).

VT/VF

Patients must have:

any of the following:

Ventricular fibrillation and flutter (UMLS:ICD10CM:I49.0); or

Ventricular tachycardia (UMLS:ICD10CM:I47.2); or

Ventricular fibrillation (UMLS:ICD10CM:I49.01).

Hosp. for HF

Patients must have:

Heart failure (UMLS:ICD10CM:I50) (principal Indicator: Primary Priority).

**ESKD**		
**Outcome definition**		
Procedure	UMLS:CPT:1012740	Dialysis Services and Procedures
Procedure	UMLS:CPT:90945	Dialysis procedure other than hemodialysis (eg, peritoneal dialysis, hemofiltration, or other continuous renal replacement therapies), with single evaluation by a physician or other qualified health care professional
Procedure	UMLS:CPT:1029674	Dialysis Circuit Procedures
Procedure	UMLS:CPT:90947	Dialysis procedure other than hemodialysis (eg, peritoneal dialysis, hemofiltration, or other continuous renal replacement therapies) requiring repeated evaluations by a physician or other qualified health care professional, with or without substantial revision of dialysis prescription
Procedure	UMLS:SNOMED:108241001	Dialysis procedure
Procedure	UMLS:CPT:1008098	Renal Transplantation Procedures
Procedure	UMLS:ICD10PCS:0TY00Z0	Transplantation of Right Kidney, Allogeneic, Open Approach
Procedure	UMLS:ICD10PCS:0TY10Z0	Transplantation of Left Kidney, Allogeneic, Open Approach
Procedure	UMLS:ICD10PCS:0TY10Z1	Transplantation of Left Kidney, Syngeneic, Open Approach
Procedure	UMLS:ICD10PCS:0TY10Z2	Transplantation of Left Kidney, Zooplastic, Open Approach
Procedure	UMLS:ICD10PCS:0TY00Z1	Transplantation of Right Kidney, Syngeneic, Open Approach
Procedure	UMLS:ICD10PCS:0TY00Z2	Transplantation of Right Kidney, Zooplastic, Open Approach
Procedure	UMLS:ICD10PCS:0TY0	Urinary System / Transplantation / Kidney, Right
Procedure	UMLS:ICD10PCS:0TY1	Urinary System / Transplantation / Kidney, Left
**Settings for the performed analyses**		
Risk analysis		excluding patients with outcome prior to the time window
Kaplan - Meier survival analysis		excluding patients with outcome prior to the time window

Presbyopia

Patients must have:

Presbyopia (UMLS:ICD10CM:H52.4).

Cataract surgery

Patients must have:

any of the following:

Cataract surgery (UMLS:SNOMED:110473004); or

Cataract extraction status (UMLS:ICD10CM:Z98.4).

Supplemental Table 3 - Baseline characteristics in men before and after propensity score matching

	Before			After		
	**Topiramate**	**No Topiramate**	**Std diff. (%)**	**Topiramate**	**No Topiramate**	**Std diff. (%)**
	**n = 41,510**	**n = 292,317**		**n = 41,503**	**n = 41,503**	
**Clinical characteristics**						
Age at Index, n (%)	44.5 +/- 15.0	44.8 +/- 16.5	1.5	44.5 +/- 15.0	44.8 +/- 15.5	1.8
Men, n (%)	41,510 (100%)	292,317 (100%)	0	41,503 (100%)	41,503 (100%)	0
White, n (%)	29,888 (72%)	204,696 (70%)	4.4	29,884 (72%)	29,692 (71.5%)	1
Black or African American, n (%)	5,056 (12.2%)	33,658 (11.5%)	2.1	5,055 (12.2%)	4,815 (11.6%)	1.8
Hispanic or Latino, n (%)	2,829 (6.8%)	19,164 (6.6%)	1	2,829 (6.8%)	2,768 (6.7%)	0.6
Asian, n (%)	764 (1.8%)	9,549 (3.3%)	9	764 (1.8%)	751 (1.8%)	0.2
Systolic BP (mm Hg), mean±SD	126.6 +/- 16.2	127.3 +/- 17.2	4	126.6 +/- 16.2	127.3 +/- 17.3	4
Diastolic BP (mm Hg), mean±SD	77.5 +/- 11.2	77.3 +/- 11.8	1.7	77.5 +/- 11.2	77.5 +/- 11.8	0
Body mass index (kg/m^2), mean±SD	30.5 +/- 7.3	28.6 +/- 6.3	28.4	30.5 +/- 7.3	29.9 +/- 6.8	8.7
**Comorbid conditions**						
Hypertension, n (%)	13,573 (32.7%)	90,759 (31%)	3.5	13,570 (32.7%)	13,351 (32.2%)	1.1
Diabetes mellitus, n (%)	5,280 (12.7%)	31,789 (10.9%)	5.7	5,277 (12.7%)	5,111 (12.3%)	1.2
Smoker, n (%)	3,646 (8.8%)	24,409 (8.4%)	1.5	3,646 (8.8%)	3,562 (8.6%)	0.7
Overweight or obesity, n (%)	7,427 (17.9%)	38,677 (13.2%)	12.9	7,424 (17.9%)	7,354 (17.7%)	0.4
Dyslipidaemia, n (%)	11,945 (28.8%)	82,596 (28.3%)	1.2	11,945 (28.8%)	11,716 (28.2%)	1.2
Alcohol related diagnoses, n (%)	1,594 (3.8%)	8,729 (3%)	4.7	1,593 (3.8%)	1,573 (3.8%)	0.3
Heart failure, n (%)	1,543 (3.7%)	9,855 (3.4%)	1.9	1,543 (3.7%)	1,488 (3.6%)	0.7
Coronary artery disease, n (%)	3,454 (8.3%)	23,339 (8%)	1.2	3,454 (8.3%)	3,421 (8.2%)	0.3
Myocardial infarction, n (%)	747 (1.8%)	5,604 (1.9%)	0.9	747 (1.8%)	1,009 (2.4%)	4.4
Dilated cardiomyopathy, n (%)	122 (0.3%)	923 (0.3%)	0.4	122 (0.3%)	121 (0.3%)	0

Ischemic stroke, n (%)	1,871 (4.5%)	10,238 (3.5%)	5.1	1,869 (4.5%)	1,819 (4.4%)	0.6
Intracranial hemorrhage, n (%)	357 (0.9%)	1,671 (0.6%)	3.4	357 (0.9%)	315 (0.8%)	1.1
Valve disease, n (%)	918 (2.2%)	7,131 (2.4%)	1.5	918 (2.2%)	934 (2.3%)	0.3
Atrial fibrillation or flutter, n (%)	1,538 (3.7%)	12,093 (4.1%)	2.2	1,538 (3.7%)	1,487 (3.6%)	0.7
Previous ICD, n (%)	264 (0.6%)	1,427 (0.5%)	2	264 (0.6%)	202 (0.5%)	2
Kidney disease, n (%)	2,849 (6.9%)	19,613 (6.7%)	0.6	2,849 (6.9%)	2,804 (6.8%)	0.4
Lung disease, n (%)	17,431 (42%)	118,578 (40.6%)	2.9	17,428 (42%)	16,803 (40.5%)	3.1
COPD, n (%)	1,850 (4.5%)	10,454 (3.6%)	4.5	1,850 (4.5%)	1,793 (4.3%)	0.7
Sleep apnoea syndrome, n (%)	7,158 (17.2%)	35,285 (12.1%)	14.7	7,156 (17.2%)	7,002 (16.9%)	1
Peripheral vascular disease, n (%)	746 (1.8%)	4,438 (1.5%)	2.2	746 (1.8%)	737 (1.8%)	0.2
Previous cancer, n (%)	6,904 (16.6%)	51,234 (17.5%)	2.4	6,904 (16.6%)	6,790 (16.4%)	0.7
Thyroid diseases, n (%)	2,906 (7%)	17,823 (6.1%)	3.7	2,906 (7%)	2,549 (6.1%)	3.5
Malnutrition, n (%)	615 (1.5%)	4,514 (1.5%)	0.5	615 (1.5%)	709 (1.7%)	1.8
Cognitive impairment, n (%)	44 (0.1%)	395 (0.1%)	0.8	44 (0.1%)	46 (0.1%)	0.1
Anxiety, n (%)	12,143 (29.3%)	67,810 (23.2%)	13.8	12,138 (29.2%)	11,912 (28.7%)	1.2
Depression / Mood disorders, n (%)	11,396 (27.5%)	57,681 (19.7%)	18.3	11,393 (27.5%)	11,141 (26.8%)	1.4
**Biological characteristics**						
Total cholesterol (mg/dL), mean±SD	176.2 +/- 47.6	177.1 +/- 45.8	1.9	176.3 +/- 47.6	176.8 +/- 47.1	1.1
LDL cholesterol (mg/dL), mean±SD	103.5 +/- 38.1	104.6 +/- 37.4	2.7	103.5 +/- 38.1	103.6 +/- 37.8	0.1
HDL cholesterol (mg/dL), mean±SD	42.5 +/- 15.4	44.9 +/- 15.5	15.9	42.5 +/- 15.4	43.5 +/- 15.3	7
Triglyceride (mg/dL), mean±SD	162.0 +/- 153.1	145.9 +/- 140.5	11	162.0 +/- 153.1	156.7 +/- 144.4	3.6
Hemoglobin A1c (%), mean±SD	6.0 +/- 1.6	6.0 +/- 1.5	0.4	6.0 +/- 1.6	6.1 +/- 1.6	5.7
Estimated GFR (MDRD, ml/min), mean±SD	87.2 +/- 24.9	86.2 +/- 25.5	3.8	87.2 +/- 24.9	86.6 +/- 25.8	2.2
Albuminuria 0-30 mg/g, n (%)	235 (0.6%)	1,869 (0.6%)	0.9	235 (0.6%)	254 (0.6%)	0.6
Albuminuria 30-300 mg/g, n (%)	76 (0.2%)	592 (0.2%)	0.4	76 (0.2%)	73 (0.2%)	0.2
Albuminuria >300 mg/g, n (%)	25 (0.1%)	293 (0.1%)	1.4	25 (0.1%)	29 (0.1%)	0.4
**Treatments**						

Beta Blockers, n (%)	12,575 (30.3%)	54,874 (18.8%)	27	12,568 (30.3%)	12,682 (30.6%)	0.6
Calcium Channel Blockers, n (%)	7,628 (18.4%)	32,616 (11.2%)	20.5	7,623 (18.4%)	7,577 (18.3%)	0.3
ACE Inhibitors, n (%)	6,939 (16.7%)	31,396 (10.7%)	17.4	6,932 (16.7%)	6,790 (16.4%)	0.9
Angiotensin II Inhibitors, n (%)	4,014 (9.7%)	18,974 (6.5%)	11.7	4,011 (9.7%)	3,950 (9.5%)	0.5
Digitalis glycosides, n (%)	241 (0.6%)	1,291 (0.4%)	1.9	241 (0.6%)	217 (0.5%)	0.8
Diuretics, n (%)	7,752 (18.7%)	34,327 (11.7%)	19.4	7,746 (18.7%)	7,647 (18.4%)	0.6
Lipid lowering drugs, n (%)	11,995 (28.9%)	54,392 (18.6%)	24.4	11,989 (28.9%)	11,961 (28.8%)	0.1
Glucose-lowering therapy, n (%)	6,285 (15.1%)	27,059 (9.3%)	18.1	6,278 (15.1%)	6,090 (14.7%)	1.3
Insulin, n (%)	3,883 (9.4%)	17,822 (6.1%)	12.2	3,882 (9.4%)	3,819 (9.2%)	0.5
Non-insulin glucose-lowering therapy, n (%)	4,410 (10.6%)	17,087 (5.8%)	17.4	4,403 (10.6%)	4,110 (9.9%)	2.3
Metformin, n (%)	3,497 (8.4%)	13,705 (4.7%)	15.1	3,493 (8.4%)	3,387 (8.2%)	0.9
Sulfonylureas, n (%)	1,411 (3.4%)	5,283 (1.8%)	10	1,406 (3.4%)	1,254 (3%)	2.1
GLP-1 receptor agonists, n (%)	869 (2.1%)	3,027 (1%)	8.5	867 (2.1%)	847 (2%)	0.3
DPP4 inhibitors, n (%)	661 (1.6%)	2,789 (1%)	5.7	661 (1.6%)	628 (1.5%)	0.6
SGLT2 inhibitors, n (%)	663 (1.6%)	2,721 (0.9%)	6	662 (1.6%)	669 (1.6%)	0.1
Thiazolidinediones, n (%)	359 (0.9%)	1,267 (0.4%)	5.4	358 (0.9%)	270 (0.7%)	2.4
Antiplatelet therapy, n (%)	9,576 (23.1%)	46,287 (15.8%)	18.4	9,571 (23.1%)	9,688 (23.3%)	0.7
Anticoagulant, n (%)	8,659 (20.9%)	43,221 (14.8%)	15.9	8,656 (20.9%)	8,704 (21%)	0.3

SD: standard deviation, ICD: implantable cardioverter defibrillator, BP: blood pressure, COPD: chronic obstructive pulmonary disease, GFR: glomerular filtration rate, ACE: angiotensin-converting enzyme, GLP1: glucagon-like protein, DPP4: dipeptyl peptidase, SGLT2: sodium-glucose transport protein-2

Supplemental Table 4 - Baseline characteristics in women before and after propensity score matching

	Before			After		
	**Topiramate**	**No Topiramate**	**Std diff. (%)**	**Topiramate**	**No Topiramate**	**Std diff. (%)**
	**n = 247,535**	**n = 1,123,952**		**n = 247,253**	**n = 247,253**	
**Clinical characteristics**						
Age at Index, n (%)	42.1 +/- 14.0	42.5 +/- 15.9	2.4	42.1 +/- 14.0	42.4 +/- 14.7	2.3
Men, n (%)	0 (0%)	0 (0%)	0	0 (0%)	0 (0%)	0
White, n (%)	171,505 (69.3%)	761,276 (67.7%)	3.3	171,338 (69.3%)	168,754 (68.3%)	2.3
Black or African American, n (%)	37,244 (15%)	145,109 (12.9%)	6.2	37,155 (15%)	35,646 (14.4%)	1.7
Hispanic or Latino, n (%)	19,068 (7.7%)	89,306 (7.9%)	0.9	19,051 (7.7%)	19,657 (8%)	0.9
Asian, n (%)	3,856 (1.6%)	37,057 (3.3%)	11.3	3,856 (1.6%)	3,731 (1.5%)	0.4
Systolic BP (mm Hg), mean±SD	121.3 +/- 16.5	121.7 +/- 17.4	2.7	121.3 +/- 16.5	122.3 +/- 17.5	5.7
Diastolic BP (mm Hg), mean±SD	74.9 +/- 11.3	74.3 +/- 11.6	5.3	74.9 +/- 11.3	74.7 +/- 11.7	1
Body mass index (kg/m2), mean±SD	31.3 +/- 8.6	29.0 +/- 7.7	28.9	31.3 +/- 8.6	30.8 +/- 8.3	6.7
**Comorbid conditions**						
Hypertension, n (%)	56,110 (22.7%)	247,160 (22%)	1.6	55,991 (22.6%)	54,967 (22.2%)	1
Diabetes mellitus, n (%)	22,805 (9.2%)	89,202 (7.9%)	4.6	22,748 (9.2%)	22,433 (9.1%)	0.4
Smoker, n (%)	14,701 (5.9%)	63,474 (5.6%)	1.2	14,676 (5.9%)	14,529 (5.9%)	0.3
Overweight or obesity, n (%)	53,533 (21.6%)	172,611 (15.4%)	16.2	53,366 (21.6%)	52,601 (21.3%)	0.8
Dyslipidaemia, n (%)	47,703 (19.3%)	214,407 (19.1%)	0.5	47,606 (19.3%)	47,036 (19%)	0.6
Alcohol related diagnoses, n (%)	3,501 (1.4%)	11,488 (1%)	3.6	3,483 (1.4%)	3,441 (1.4%)	0.1
Heart failure, n (%)	5,070 (2%)	20,804 (1.9%)	1.4	5,060 (2%)	4,966 (2%)	0.3
Coronary artery disease, n (%)	8,111 (3.3%)	36,186 (3.2%)	0.3	8,098 (3.3%)	8,052 (3.3%)	0.1
Myocardial infarction, n (%)	1,933 (0.8%)	8,976 (0.8%)	0.2	1,927 (0.8%)	2,451 (1%)	2.3
Dilated cardiomyopathy, n (%)	288 (0.1%)	1,227 (0.1%)	0.2	288 (0.1%)	299 (0.1%)	0.1
Ischemic stroke, n (%)	6,570 (2.7%)	23,150 (2.1%)	3.9	6,547 (2.6%)	6,354 (2.6%)	0.5
Intracranial hemorrhage, n (%)	969 (0.4%)	3,161 (0.3%)	1.9	966 (0.4%)	904 (0.4%)	0.4
Valve disease, n (%)	5,421 (2.2%)	25,409 (2.3%)	0.5	5,413 (2.2%)	5,205 (2.1%)	0.6

Atrial fibrillation or flutter, n (%)	3,591 (1.5%)	19,445 (1.7%)	2.2	3,591 (1.5%)	3,606 (1.5%)	0.1
Previous ICD, n (%)	503 (0.2%)	1,791 (0.2%)	1	502 (0.2%)	486 (0.2%)	0.1
Kidney disease, n (%)	8,811 (3.6%)	37,474 (3.3%)	1.2	8,799 (3.6%)	8,781 (3.6%)	0
Lung disease, n (%)	108,511 (43.8%)	466,659 (41.5%)	4.7	108,355 (43.8%)	105,075 (42.5%)	2.7
COPD, n (%)	7,444 (3%)	27,865 (2.5%)	3.2	7,422 (3%)	7,271 (2.9%)	0.4
Sleep apnoea syndrome, n (%)	22,825 (9.2%)	66,096 (5.9%)	12.7	22,704 (9.2%)	22,221 (9%)	0.7
Peripheral vascular disease, n (%)	2,108 (0.9%)	8,921 (0.8%)	0.6	2,101 (0.8%)	2,307 (0.9%)	0.9
Previous cancer, n (%)	43,846 (17.7%)	196,950 (17.5%)	0.5	43,777 (17.7%)	43,493 (17.6%)	0.3
Thyroid diseases, n (%)	36,282 (14.7%)	162,120 (14.4%)	0.7	36,222 (14.6%)	37,739 (15.3%)	1.7
Malnutrition, n (%)	2,578 (1%)	11,211 (1%)	0.4	2,570 (1%)	2,918 (1.2%)	1.3
Cognitive impairment, n (%)	197 (0.1%)	1,238 (0.1%)	1	197 (0.1%)	191 (0.1%)	0.1
Anxiety, n (%)	83,432 (33.7%)	317,482 (28.2%)	11.8	83,281 (33.7%)	81,630 (33%)	1.4
Depression / Mood disorders, n (%)	79,731 (32.2%)	272,818 (24.3%)	17.7	79,549 (32.2%)	77,774 (31.5%)	1.5
**Biological characteristics**						
Total cholesterol (mg/dL), mean±SD	184.7 +/- 45.8	187.0 +/- 44.0	5	184.7 +/- 45.8	185.4 +/- 44.3	1.5
LDL cholesterol (mg/dL), mean±SD	107.5 +/- 36.4	107.6 +/- 35.7	0.2	107.5 +/- 36.4	107.3 +/- 36.0	0.6
HDL cholesterol (mg/dL), mean±SD	52.1 +/- 18.9	55.5 +/- 19.6	17.4	52.1 +/- 18.9	52.9 +/- 19.3	4
Triglyceride (mg/dL), mean±SD	128.5 +/- 110.8	120.5 +/- 105.9	7.5	128.5 +/- 110.8	127.5 +/- 133.0	0.8
Hemoglobin A1c (%), mean±SD	5.8 +/- 1.4	5.8 +/- 1.4	0.6	5.8 +/- 1.4	5.8 +/- 1.4	3.5
Estimated GFR (ml/min), mean±SD	87.1 +/- 25.0	89.4 +/- 27.5	8.7	87.1 +/- 25.0	88.0 +/- 27.2	3.3
Albuminuria 0-30 mg/g, n (%)	1,107 (0.4%)	5,650 (0.5%)	0.8	1,106 (0.4%)	1,044 (0.4%)	0.4
Albuminuria 30-300 mg/g, n (%)	304 (0.1%)	1,668 (0.1%)	0.7	304 (0.1%)	309 (0.1%)	0.1
Albuminuria >300 mg/g, n (%)	111 (0%)	737 (0.1%)	0.9	111 (0%)	111 (0%)	0
**Treatments**						
Beta Blockers, n (%)	60,509 (24.4%)	166,959 (14.9%)	24.3	60,241 (24.4%)	61,788 (25%)	1.5
Calcium Channel Blockers, n (%)	29,830 (12.1%)	84,237 (7.5%)	15.4	29,645 (12%)	29,986 (12.1%)	0.4
ACE Inhibitors, n (%)	22,513 (9.1%)	64,670 (5.8%)	12.8	22,349 (9%)	22,002 (8.9%)	0.5
Angiotensin II Inhibitors, n (%)	15,563 (6.3%)	49,532 (4.4%)	8.4	15,482 (6.3%)	15,394 (6.2%)	0.1
Digitalis glycosides, n (%)	652 (0.3%)	2,565 (0.2%)	0.7	649 (0.3%)	724 (0.3%)	0.6

Diuretics, n (%)	44,931 (18.2%)	119,331 (10.6%)	21.6	44,678 (18.1%)	44,778 (18.1%)	0.1
Lipid lowering drugs, n (%)	43,791 (17.7%)	124,503 (11.1%)	18.9	43,537 (17.6%)	43,457 (17.6%)	0.1
Glucose-lowering therapy, n (%)	31,420 (12.7%)	79,838 (7.1%)	18.8	31,191 (12.6%)	30,388 (12.3%)	1
Insulin, n (%)	15,865 (6.4%)	43,983 (3.9%)	11.3	15,776 (6.4%)	15,379 (6.2%)	0.7
Non-insulin glucose-lowering therapy, n (%)	23,383 (9.4%)	55,657 (5%)	17.5	23,178 (9.4%)	22,274 (9%)	1.3
Metformin, n (%)	18,287 (7.4%)	43,642 (3.9%)	15.2	18,133 (7.3%)	17,661 (7.1%)	0.7
Sulfonylureas, n (%)	4,783 (1.9%)	12,390 (1.1%)	6.8	4,721 (1.9%)	4,443 (1.8%)	0.8
GLP-1 receptor agonists, n (%)	5,791 (2.3%)	11,963 (1.1%)	9.9	5,721 (2.3%)	5,462 (2.2%)	0.7
DPP4 inhibitors, n (%)	2,431 (1%)	6,928 (0.6%)	4.1	2,427 (1%)	2,358 (1%)	0.3
SGLT2 inhibitors, n (%)	1,964 (0.8%)	5,425 (0.5%)	3.9	1,953 (0.8%)	1,893 (0.8%)	0.3
Thiazolidinediones, n (%)	1,075 (0.4%)	2,608 (0.2%)	3.5	1,058 (0.4%)	964 (0.4%)	0.6
Antiplatelet therapy, n (%)	40,251 (16.3%)	120,637 (10.7%)	16.2	40,072 (16.2%)	40,981 (16.6%)	1
Anticoagulant, n (%)	41,215 (16.7%)	122,048 (10.9%)	16.9	41,021 (16.6%)	41,302 (16.7%)	0.3

SD: standard deviation, ICD: implantable cardioverter defibrillator, BP: blood pressure, COPD: chronic obstructive pulmonary disease, GFR: glomerular filtration rate, ACE: angiotensin-converting enzyme, GLP1: glucagon-like protein, DPP4: dipeptyl peptidase, SGLT2: sodium-glucose transport protein-2.

Supplemental Table 5. Clinical outcomes during follow-up in the unmatched population (3.4 +/- 2.3 years)

	Topiramate (n = 323,877)		No Topiramate (n = 1,510,232)			
	**Number of events**	**Yearly rate, %**	**Number of events**	**Yearly rate, %**	**Hazard ratio (95% CI)**	**p value**
**Death**	7576	0.60	42692	0.77	0.742 (0.724-0.760)	<0.0001
**Chronic dialysis or renal transplantation**	854	0.07	4248	0.08	0.85 (0.789-0.915)	<0.0001
**Acute MI**	4006	0.34	15693	0.31	1.068 (1.031-1.105)	<0.0001
**Ischemic stroke or thromboembolism**	7983	0.65	24939	0.47	1.368 (1.334-1.403)	<0.0001
**AF**	5336	0.44	21934	0.43	1.016 (0.986-1.047)	0.29
**VT/VF/Cardiac arrest**	3827	0.30	15120	0.27	1.069 (1.031-1.107)	<0.0001
**Hosp. for HF**	2334	0.17	9709	0.16	1.031 (0.985-1.078)	0.19
**Presbyopia**	10448	0.82	37638	0.68	1.169 (1.144-1.195)	<0.0001
**Cataract surgery**	1543	0.13	7677	0.14	0.843 (0.798-0.890)	<0.0001

Chronic dialysis: based on diagnostic codes (not dialysis sessions).

Supplementary Table 6. Summary of GFR and albuminuria changes (for patients having both baseline and 24-36 months follow-up evaluations) with paired and independent t-test results

Measure	Group	n	Baseline (Mean ± SD)	Follow-up (Mean ± SD)	Δ (Change)	p-value
**GFR**	**Topiramate**	81,407	79.9 ± 24.0	79.3 ± 25.3	-0.6	< 0.0001
	**No Topiramate**	81,407	80.7 ± 24.7	79.8 ± 26.1	-0.9	< 0.0001
	**Topiramate vs No Topiramate**				-0.3	0.035
**Albuminuria**	**Topiramate**	697	68 ± 307	79 ± 293	11.2	0.2
	**No Topiramate**	697	97 ± 386	169 ± 600	72.2	< 0.0001
	**Topiramate vs No Topiramate**				61.0	0.001

Supplemental Figure 1: Covariate balance before and after propensity score matching

Red symbol: before propensity score matching. Blue symbol: after propensity score matching.

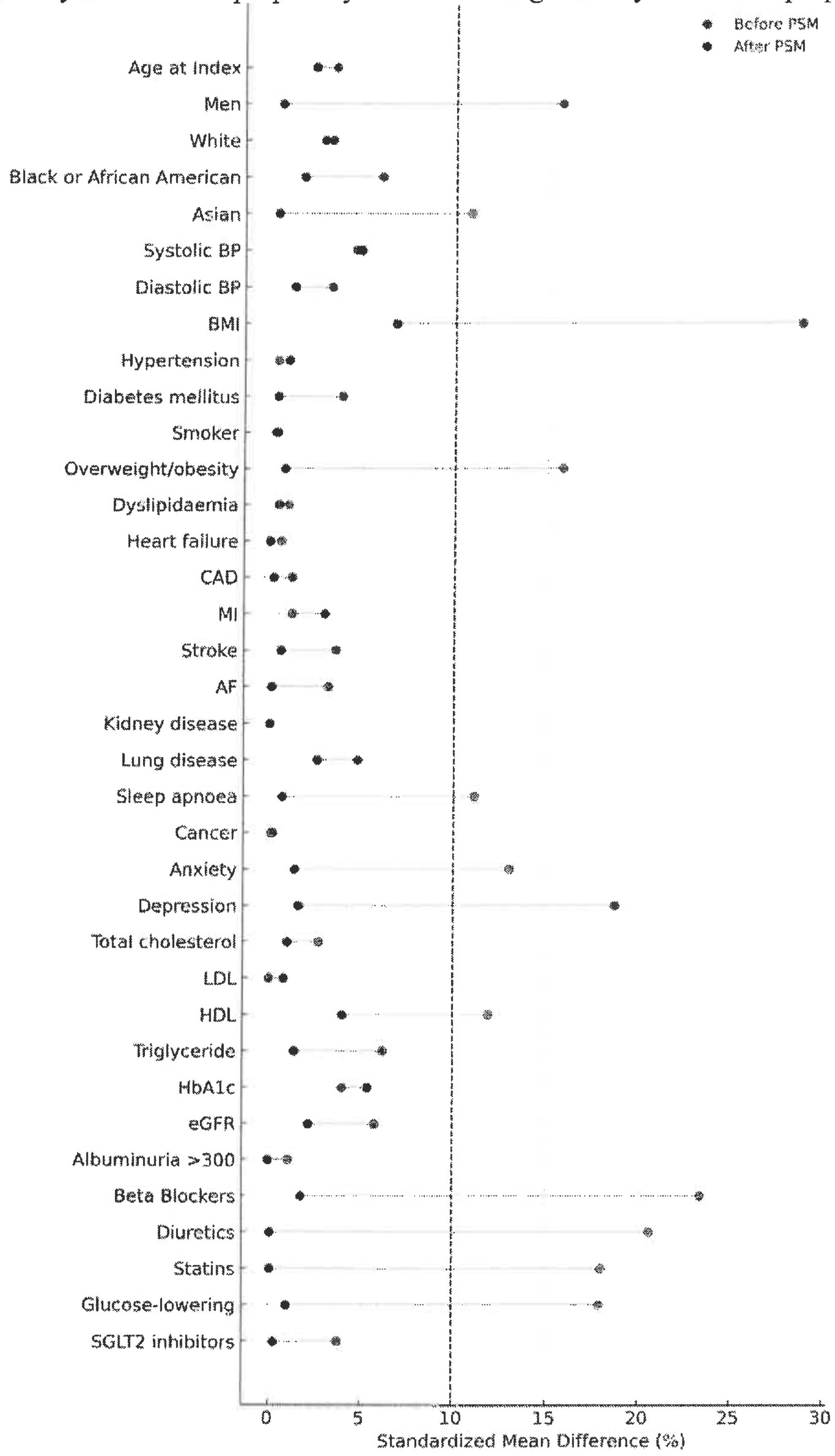

Supplemental Figure 2. Propensity score density function in all patients - Before (left panel) and after matching (right panel).

1 - Migraine, Topiramate	295,560	291,728
2 - Migraine, No Topiramate	1,495,601	291,728

Propensity score density function - Before and after matching (topiramate - purple, No Topiramate - green)

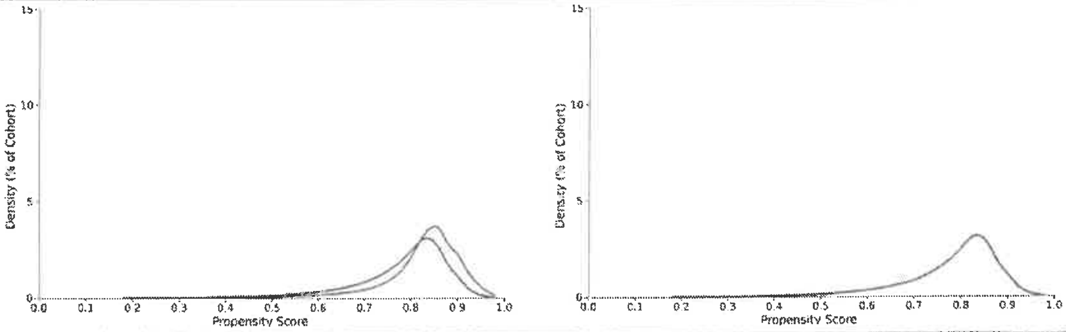

Topiramate (N = 295,560) and No Topiramte (N = 1,495,601) characteristics before propensity score matching

Supplemental Figure 3 - Propensity score density function in men - Before (left panel) and after matching (right panel).

**Cohort 1 and cohort 2 patient count before and after propensity score matching**		
Cohort	Patient count before matching	Patient count after matching
1 - Migraine, Topiramate	41,975	41,503
2 - Migraine, No Topiramate	300,951	41,503

Propensity score density function - Before and after matching (Topiramate - purple, No Topiramate - green)

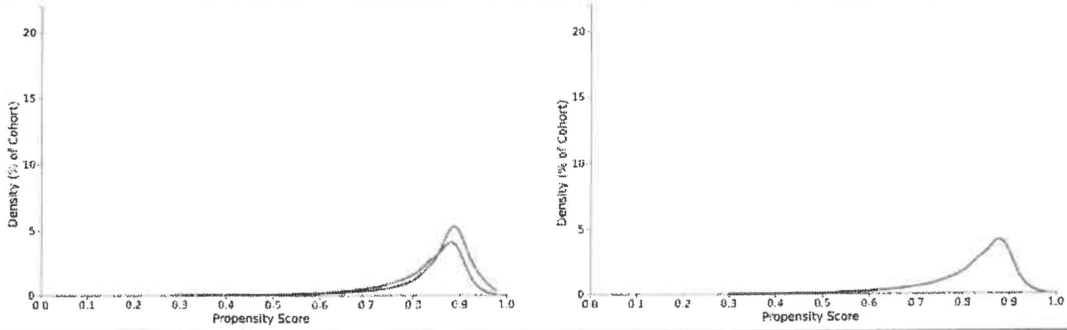

Supplemental Figure 4 - Propensity score density function in women - Before (left panel) and after matching (right panel).

Cohort 1 and cohort 2 patient count before and after propensity score matching

Cohort	Patient count before matching	Patient count after matching
1 - Migraine, Topiramate	250,648	247,253
2 - Migraine, No Topiramate	1,157,574	247,253

Propensity score density function - Before and after matching (Topiramate - purple, No topiramate - green)

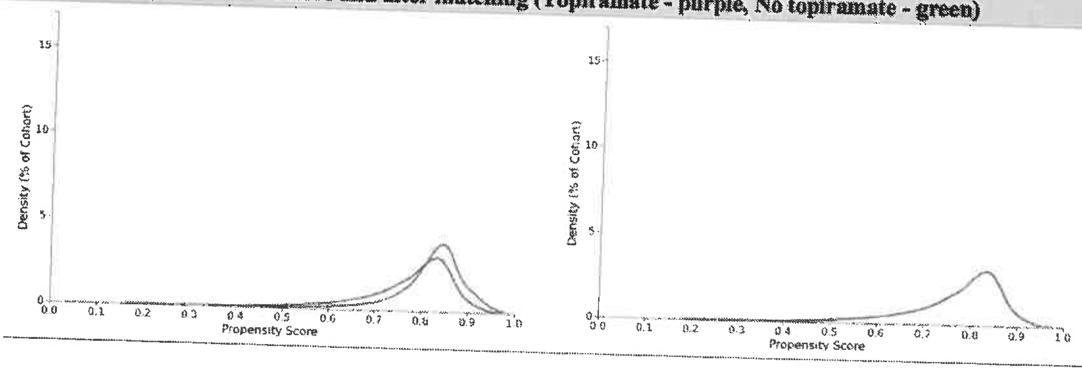

STROBE Statement—checklist of items that should be included in reports of observational studies

	Item No	Recommendation
**Title and abstract**	1	(*a*) Indicate the study's design with a commonly used term in the title or the abstract
		(*b*) Provide in the abstract an informative and balanced summary of what was done and what was found
**Introduction**		
Background/rationale	2	Explain the scientific background and rationale for the investigation being reported
Objectives	3	State specific objectives, including any prespecified hypotheses
**Methods**		
Study design	4	Present key elements of study design early in the paper
Setting	5	Describe the setting, locations, and relevant dates, including periods of recruitment, exposure, follow-up, and data collection
Participants	6	(*a*) *Cohort study*—Give the eligibility criteria, and the sources and methods of selection of participants. Describe methods of follow-up<br>*Case-control study*—Give the eligibility criteria, and the sources and methods of case ascertainment and control selection. Give the rationale for the choice of cases and controls<br>*Cross-sectional study*—Give the eligibility criteria, and the sources and methods of selection of participants
		(*b*) *Cohort study*—For matched studies, give matching criteria and number of exposed and unexposed<br>*Case-control study*—For matched studies, give matching criteria and the number of controls per case
Variables	7	Clearly define all outcomes, exposures, predictors, potential confounders, and effect modifiers. Give diagnostic criteria, if applicable
Data sources/ measurement	8*	For each variable of interest, give sources of data and details of methods of assessment (measurement). Describe comparability of assessment methods if there is more than one group
Bias	9	Describe any efforts to address potential sources of bias
Study size	10	Explain how the study size was arrived at
Quantitative variables	11	Explain how quantitative variables were handled in the analyses. If applicable, describe which groupings were chosen and why
Statistical methods	12	(*a*) Describe all statistical methods, including those used to control for confounding
		(*b*) Describe any methods used to examine subgroups and interactions
		(*c*) Explain how missing data were addressed
		(*d*) *Cohort study*—If applicable, explain how loss to follow-up was addressed<br>*Case-control study*—If applicable, explain how matching of cases and controls was addressed<br>*Cross-sectional study*—If applicable, describe analytical methods taking account of sampling strategy
		(*e*) Describe any sensitivity analyses

Continued on next page

**Results**		
Participants	13*	(a) Report numbers of individuals at each stage of study—eg numbers potentially eligible, examined for eligibility, confirmed eligible, included in the study, completing follow-up, and analysed
		(b) Give reasons for non-participation at each stage
		(c) Consider use of a flow diagram
Descriptive data	14*	(a) Give characteristics of study participants (eg demographic, clinical, social) and information on exposures and potential confounders
		(b) Indicate number of participants with missing data for each variable of interest
		(c) *Cohort study*—Summarise follow-up time (eg, average and total amount)
Outcome data	15*	*Cohort study*—Report numbers of outcome events or summary measures over time
		Case-control study—Report numbers in each exposure category, or summary measures of exposure
		Cross-sectional study—Report numbers of outcome events or summary measures
Main results	16	(*a*) Give unadjusted estimates and, if applicable, confounder-adjusted estimates and their precision (eg, 95% confidence interval). Make clear which confounders were adjusted for and why they were included
		(*b*) Report category boundaries when continuous variables were categorized
		(*c*) If relevant, consider translating estimates of relative risk into absolute risk for a meaningful time period
Other analyses	17	Report other analyses done—eg analyses of subgroups and interactions, and sensitivity analyses
**Discussion**		
Key results	18	Summarise key results with reference to study objectives
Limitations	19	Discuss limitations of the study, taking into account sources of potential bias or imprecision. Discuss both direction and magnitude of any potential bias
Interpretation	20	Give a cautious overall interpretation of results considering objectives, limitations, multiplicity of analyses, results from similar studies, and other relevant evidence
Generalisability	21	Discuss the generalisability (external validity) of the study results
**Other information**		
Funding	22	Give the source of funding and the role of the funders for the present study and, if applicable, for the original study on which the present article is based

*Give information separately for cases and controls in case-control studies and, if applicable, for exposed and unexposed groups in cohort and cross-sectional studies.

Note: An Explanation and Elaboration article discusses each checklist item and gives methodological background and published examples of transparent reporting. The STROBE checklist is best used in conjunction with this article (freely available on the Web sites of PLoS Medicine at http://www.plosmedicine.org/, Annals of Internal Medicine at http://www.annals.org/, and Epidemiology at http://www.epidem.com/). Information on the STROBE Initiative is available at www.strobe-statement.org.
